# AmMYB24 Regulates Floral Terpenoid Biosynthesis Induced by Blue Light in Snapdragon Flowers

**DOI:** 10.3389/fpls.2022.885168

**Published:** 2022-07-01

**Authors:** Jianing Han, Tong Li, Xuelian Wang, Xi Zhang, Xiaoning Bai, Huihui Shao, Shaojie Wang, Zenghui Hu, Jing Wu, Pingsheng Leng

**Affiliations:** ^1^Beijing Advanced Innovation Center for Tree Breeding by Molecular Design, Beijing University of Agriculture, Beijing, China; ^2^College of Landscape Architecture, Beijing University of Agriculture, Beijing, China; ^3^Institute of Forestry New Technology, Chinese Academy of Forestry, Beijing, China

**Keywords:** blue light, floral volatile, MYB, snapdragon, terpenoid, transcription factor

## Abstract

Floral terpenoid volatiles are impacted by light quality. In snapdragon, blue light can significantly enhance the emissions of ocimene and myrcene and the expression of ocimene synthase (*AmOCS*) and myrcene synthase (*AmMYS*). However, the mechanisms underlying the response to blue light are largely unknown. In this study, two transcription factors (TFs), AmMYB24 and AmMYB63 were screened which showed high expression level under blue light. AmMYB24 exhibited synchronous expression with *AmOCS*. Moreover, *AmOCS* transcript expression was up-regulated in response to AmMYB24 overexpression. This activation is direct and occurs through binding of AmMYB24 to MYBCORECYCATB1 sites in the *AmOCS* promoter. In addition, AmMYB24 interacts with the blue light signal key receptor *AmCRY1* and the transcriptional activation activity of *AmMYB24* was decreased in AmCRY1 silencing flowers. Taken together, our results revealed the regulatory pathway of biosynthesis of ocimene induced by blue light mediated by AmMYB24 and AmCRY1. When snapdragon flowers were exposed to blue light, AmCRY1 was first activated, the light signal is transduced to AmMYB24 through interaction with AmCRY1, and finally AmMYB24 activates *AmOCS* by binding to its MYBCOREATCYCB1 motif, resulting in abundant ocimene emission.

## Introduction

Floral volatiles are important plant secondary metabolites, and terpenoids are the largest category of these compounds. Floral terpenoid volatiles contain monoterpenoids and sesquiterpenoids, which are produced from the methylerythritol phosphate (MEP) pathway in plastids and the mevalonate (MVA) pathway in cytosol, respectively (Muhlemann et al., 2014). These terpenoid volatiles have diverse physiological and ecological functions, including attraction of pollinators, defense against folivores and pathogens, and signaling in plant-plant interactions (Pichersky et al., 2006; Huang et al., 2012; Boncan et al., 2020), and also are widely used in the cosmetics and perfume industries and for medicinal applications (Rydén et al., 2010). Thus, there is significant interest in the regulatory mechanisms of floral terpenoid volatile biosynthesis.

For plants, light not only provides energy, but also serves as an important environmental signal that regulates plant growth and development as well as the production of secondary metabolites (Kami et al., 2010). Plants can sense and respond to a broad-spectrum of light, spanning from UV-C to the far-red, with different effects on plant growth, development, and morphology (Galvao and Fankhauser, 2015). Light quality also affects plant volatiles (Carvalho et al., 2016). In the environment, short wave light decreases with the increase of latitude, but is positively correlated with altitude. Shortwave light is more abundant in the summer when plants grow vigorously than in the winter. In addition, compared with the natural radiation from the sky, the proportion of blue light increased in the shade of buildings (Yu et al., 2015). Other than studies that detected the terpenoids emitted from leaves and fruits (Maffei and Scannerini, 1999; Arena et al., 2016; Liu et al., 2017; Wu et al., 2020), only some previous studies investigated the effect of light qualities on floral terpenoid volatiles. Red light exposure resulted in increased emission of (E)-β-Caryophyllene compared to the emission levels for plants subjected to white light and blue light (Hong et al., 2012). *Phalaenopsis violacea* ‘KHM2212’ flowers were reported to emit more monoterpenes under blue light than ones subjected to red light and dark conditions (Chuang et al., 2017). Thus, further research is required to characterize the light responses of these plants. In our previous study using snapdragon, we found that blue light enhanced the biosynthesis of monoterpenes and the expression levels of related biosynthetic genes compared to the levels under conditions of red and green light (Yang et al., 2022). However, the regulatory mechanisms remain poorly understood.

Transcriptional regulation plays a key role in biosynthesis of floral volatiles (Ramya et al., 2018). Several transcription factors (TFs) involved in terpenoid biosynthesis have been identified in different plants, including AP2/ERF, bHLH, MYB, NAC, WRKY, and bZIP (Dong et al., 2020), but most studies have focused on regulation in vegetative tissues and fruits (Colquhoun et al., 2013; Li et al., 2015; Shen S. L. et al., 2016). The transcriptional regulation of floral volatiles biosynthesis is not well understood. In Arabidopsis flowers, a basic helix-loop-helix TF, MYC2, directly binds to the promoters of two sesquiterpene synthase genes TPS21 and TPS11 and activates their expression, resulting in increased emission of sesquiterpene (Hong et al., 2012). In *Freesia hybrida*, two MYB regulators, FhMYB21L1 and FhMYB21L2, can significantly activate the expression of a terpene synthase, FhTPS1; FhMYB21L2 directly binds to the MYBCORE sites in the FhTPS1 promoter, indicating direct activation (Yang et al., 2020). MYB (Abbas et al., 2021a; Ke et al., 2021) and ARF5 (Abbas et al., 2021b) play crucial roles in the biosynthesis of floral terpenoid volatiles in *Hedychium coronarium*. Despite these studies, the effects of transcription regulatory responses to different light qualities on biosynthesis of floral terpenoid volatiles are largely unknown.

The photoreceptors that receive light signals initiate the downstream reaction, which may also act in the biosynthesis of floral volatiles in response to light qualities. Cryptochromes (CRYs) are photolyase like blue light receptors and their activities increased when plants were grown under blue light (Yu et al., 2010). CRYs mediate plant growth and development through regulation of many physiochemical processes, including circadian rhythms, stomata opening, guard cell development, root development, abiotic stress responses, cell cycles, programmed cell death, apical dominance, fruit and ovule development, and seed dormancy (Goggin et al., 2008). Though we have found that blue light increases the emission of floral terpenoid volatiles from snapdragon, a role of CRYs in this process has not been described.

Snapdragon is a model plant to explore the regulatory mechanism of floral volatile biosynthesis, and ocimene and myrcene are the main terpenoid components. Inspired by our previous finding that blue light exposure enhances the emission of ocimene and myrcene (Yang et al., 2022), we screened differentially expressed genes mediating terpenoid biosynthesis and correlated MYB TFs by RNA-seq under different light qualities to investigate the potential regulation by MYB of terpenoid biosynthesis. The interaction between CRY1 and MYB was determined as a key part of the signal transduction pathway in the terpenoid biosynthesis that is induced by blue light in snapdragon.

## Materials and Methods

### Plant Material and Growth Condition

The seeds of snapdragon, *Antirrhinum majus* ‘Maryland True Pink’ (Pan American Seed Co., West Chicago, IL, United States), were sowed in a mixed substrate of peat and vermiculite at a ratio of 2:1 (v/v) at 22°C and a 16/8 h light/dark cycle in a greenhouse at the Beijing University of Agriculture. The experiments were conducted when the snapdragon plants reached the full-bloom stage. The plants selected for experiment were placed in an artificial climate chamber under a 16/8 h light/dark and 22/19°C cycle with 40% relative humidity and 150 μmol⋅m^–2^⋅s^–1^ light intensity.

### Light Quality Parameters

To record the effects of different light quality, flowers were kept in a growth chamber using LED light source at 22°C with 150 μmol m^–2^⋅ s^–1^ light intensity. Blue light (470 nm), red light (630 nm), green light (530 nm) were set for different light quality, and the flowers under dark were used as control. The flowers were sampled after 4 h exposure for different light quality. For further blue light exposure, monochromatic blue light (470 nm) and polychromatic light without blue light (EB) were used, and flowers were sampled after exposed for 12 h.

### Collection and Determination of Floral Volatiles

Headspace solid-phase microextraction (SPME) and gas chromatography-mass spectrometry (GC-MS) technologies were combined to collect and examine the floral volatile compounds of snapdragon. Briefly, using 10 ng⋅mL^–1^ 3-octanol as the internal standard, 1 g of lobes infected was placed in an airtight bottle equipped with a 100 μM Carboxen/polydimethylsiloxane fiber (SigmaAldrich) at 25°C for 5 min, followed by 40°C for 40 min. The trapped floral scent compounds were subsequently desorbed and transferred to an Agilent 5975C GC-MS (Agilent Technologies) equipped with an HP^−1^MS fused-silica capillary column (0.25 mm diameter, 30 m length, and 0.25 μm film thickness) with helium as the carrier gas. The desorption program was designed as follows:

Isothermal column temperature at 40°C for 3 min, then the temperature was increased at a rate of 5°C min^−1^ to 120°C for 1 min, and the temperature was then further increased at a rate of 10°C min^–1^ to 180°C for 3 min, and then increased to 280°C for 1 min at a rate of 10°C min^–1^. The volatiles were identified by comparing the mass spectra and retention time with the NIST 2008 mass spectra library and standard samples. Total ion chromatogram (TIC) was analyzed to detect flower volatiles.

### RNA Extraction, Real-Time Quantitative PCR and Transcriptome Analysis

Total RNA was isolated from snapdragon flowers using the TransZol Up Plus RNA Kit (TransGen Biotech, Co., Ltd., Beijing, China) and subsequently reverse transcribed into cDNA using the Evo MMLV RT for PCR Kit (Accurate Biotechnology, Co., Ltd., Hunan, China) according to the manufacturer’s specifications. The TF genes and the transcript levels of putative genes in different light conditions were identified according to the snapdragon reference genome (Li et al., 2019). SYBR Green-based real-time quantitative PCR (qRT-PCR) assay was performed. The expression level of *AmUBI* (ubiquitin) gene was used as an internal control (Manchado-Rojo et al., 2012). Relative gene expression was calculated using the 2^−ΔΔ*CT*^ formula (Livak and Schmittgen, 2001). Three biological repeats were measured for each sample. All the primers used in this study are listed in Supplementary Table 1. Snapdragon exposed with blue, red, green light (150 μmol m^–2^⋅s^––1^ PPFD) and dark for 4 h for transcriptome sequencing (Novogene, Co., Ltd., Beijing, China). Sequencing libraries were generated using NEBNext Ultra RNA Library Prep Kit for Illumina^®^ (NEB, United States) following manufacturer’s recommendations. The libraries were sequenced on an Illumina Novaseq platform and 150 bp paired-end reads were generated. Clean reads were obtained by removing adapter sequence, reads containing ploy-N and low quality reads from raw data. Reference genome^1^ and gene model annotation files were downloaded from genome website directly. Index of the reference genome was built using Hisat2 v2.0.5 and paired-end clean reads were aligned to the reference genome using Hisat2 v2.0.5. featureCounts v1.5.0-p3 was used to count the reads numbers mapped to each gene. All the TF genes and the transcript levels of putative genes in different light conditions were identified according to the snapdragon reference genome. In addition, according to the threshold (FPKM >1), the 18 TFs were screened. The heatmap was generated by TBtools.

### Subcellular Localization

The full-length CDSs of *AmMYB24* and *AmMYB63* without the stop codon were fused with GFP or mCherry by cloning into the pNC-Green-SubN vector and p2300-35S-H2B-mCherry vector. The recombinant constructs were transformed into *Agrobacterium* strain GV3101 and infiltrated into tobacco leaves. After infiltration, the tobacco plants were grown under a 16-h light/8-h dark cycle for 2 days. After incubation, subcellular localization was detected using a Confocal laser microscopy (Leica TCS SP5-II).

### Cloning of Promoters

Genomic DNA was isolated from snapdragon leaves. To amplify the promoter sequences of *AmOCS, AmMYS*, *AmDXS*, and *AmDXR*, specific primers were designed according to the Snapdragon Genome Database (see text footnote 1). Finally, the 2,000 bp upstream sequences of the intiation codon ATGs of *AmOCS*, *AmMYS*, *AmDXS*, and *AmDXR* were individually cloned into the pClone007 Vector Kit (TsingKe Biological Technology Beijing, China) and then the sequences were confirmed. The promoter sequences and the potential binding sites of regulators were further analyzed online by SOGO.^2^

### Dual-Luciferase Assays

*AmOCS*, *AmMYS*, *AmDXS*, and *AmDXR* promoters were cloned into the pNC-Green-LUC vector. The cDNA of *AmMYB24* and *AmMYB63* were cloned into the pNC-Cam1304-MCS35S vector, which carries the CaMV 35S promoter, to be used as the effector (Yan et al., 2019). The recombinant reporter vectors were transformed separately into *Agrobacterium tumefaciens* strain GV3101 with the plasmid pSoup19, and the recombinant effector vectors were transformed into GV3101. Then, the effector, reporter, and control constructs were co-transfected into *Nicotiana benthamiana* leaves. After incubation for 48 h, the leaves were exposed to a 16/8 h light/dark cycle. Relative LUC activity was calculated *via* normalization to LUC activity (Liu and Axtell, 2015), the results are shown as LUC/REN ratio. The data are presented as averages of three independent biological replicates.

### Bimolecular Fluorescence Assays

For bimolecular fluorescence complementation (BiFC) assays, the open reading frame of *AmMYB24* was cloned into pNC-BiFC-Ecn and the full-length coding region of *AmCRY1* was inserted into pNC-BiFC-Enn vector. The recombinant plasmids (pNC-BiFC-Ecn-AmMYB24 and pNC-BiFCEnn-AmCRY1) and the empty vectors were transferred into GV3101, and then combinations of the plasmids were co-transformed into *Nicotiana benthamiana* leaves. Confocal laser microscopy (Leica TCS SP5-II) was used to observe the YFP signals.

### Yeast Two-Hybrid Assay

For yeast two-hybrid (Y2H) assays, the open reading frames of *AmMYB24* and *AmCRY1* were, respectively, cloned into the pGADT7 and pGBKT7 vectors, and then Y2H assays were performed using the Matchmaker Gold Y2H system user manual (Takara, Japan). Combinations of recombinant plasmids were transferred into the yeast stain Y2H GOLD. The positive clones cultivated on SD/-Leu/-Trp agar medium plates were transferred to SD/-Leu/Trp/-His/-Ade medium plates, the working concentration of X-α-Gal was 40 mg/L. The results were observed after 3 days.

### Yeast-One-Hybrid Assays

The open reading frames of *AmMYB24* and *AmMYB63* were amplified and inserted into the pGADT7 vector. Four promoters of *AmOCS*, *AmMYS*, *AmDXS*, and *AmDXR* were ligated into the pNC-AbAi vector. Three tandem copies of the MYBCORECYCB1 motifs in the promoter of *AmOCS* were also cloned into separate pNC-AbAi vectors. *Bst*BI was used to linearize the recombinant pNC-AbAi vectors prior to transforming it into Y1H-Gold for integration. To confirmed that the plasmid integrated correctly, the Matchmaker Insert Check PCR Mix 1 (Takara, Japan) was used for a colony PCR analysis. Y1H assays were performed using the Matchmaker Gold Y1H system’s user manual (Takara, Japan). The empty vector pGADT7 with pNC-AbAi-AmOCS/AmMYS/AmDXS/AmDXR and pNC-AbAi-MYBCORECYCB1 were used as negative controls. The pGADT7 and p53-AbAi were used as the positive control. The clones were transferred to SD/-Leu/Ura medium containing 300 mg/mL AbA (Aureobasidin A) and cultivated at 28°C for 2 days after selection on the SD/-Ura plates.

### Virus-Induced Gene Silencing and Over-Expression Assays

The pNC-TRV2 and pNC-MCS35S vectors were used to generate *AmMYB24* VIGS lines and overexpression lines. In VIGS assay, virus-induced gene silencing plasmid was constructed by inserting the fragment comprising the last 200 bp of coding sequence (390 –590 bp) of *AmMYB24* and *AmCRY1* (1,821–2,021 bp) into a pNC-TRV2 vector using 5 × In-Fusion HD Enzyme Premix (Takara Bio Inc., Japan). For overexpression, the full-length *AmMYB24* was inserted into pNCMCS35S vector. The VIGS construct, pNC-TRV2- AmMYB24, pNCTRV1 and the plant over-expression construct, pNC-35S-AmMYB24, were introduced into *Agrobacterium* tumefaciens GV3101 (Weidi Biotechnology Co., Shanghai, China) for snapdragon infection. The *Agrobacterium* cells were then harvested and resuspended in inoculation buffer (10 mol⋅L^−1^ MgCl2; 10 mol⋅L^−1^ MES; 200 μmol⋅L^−1^ acetosyringone) to an *O.D*. of 1.0, and incubated at room temperature for 3 h in the dark. To prevent the petals from mechanical or physical injury, the flowers at development S2 were infected by a disposable sterile syringe without a needle. After *Agrobacterium* infiltration, plants were placed in the dark for 12 h, and then cultured for 16 h under light and 8 h under dark. After 5–7 days, samples were collected for data analysis.

### Statistical Analysis

All experiments were conducted in triplicate. The data were processed using SPSS 16.0. The mean values were compared *via* one-way ANOVA. The *p*-values were evaluated using the Tukey’s multiple range test. All the parameters used in Turke’s test were default parameters in SPSS. Stars indicate the level of significance, *0.01 < *p* < 0.05, and ^**^p < 0.01.

### Accession Numbers

Sequence data from this article can be found in the Snapdragon databases (see text footnote 1) under the following accession numbers: AmMYB24 (Am06g35820) and AmMYB63 (Am07g00460).

## Results

### The Effect of Blue Light on the Expression of Terpenoid Biosynthesis-Related Genes

**FIGURE 1:**
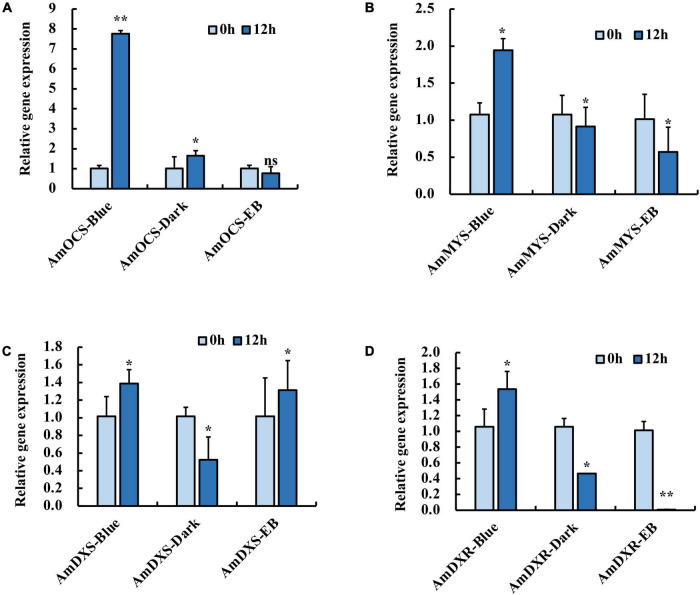
Expression profiles of **(A)** *AmOCS* **(B)** *AmMYS* **(C)** *AmDXS* and **(D)** *AmDXR* under monochromatic blue light (470 nm), polychromatic light without blue light (EB) and dark for 0 and 12 h. Each experiment was conducted with three biological replicates. The *p*-values were evaluated using the Tukey’s multiple range test. Stars indicate the level of significance, *0.01 < *p* < 0.05, and **p < 0.01.

In our previous study, blue light exposure increased expression levels of *AmOCS* and *AmMYS* more than red and green light exposure, concurrently with the emission of ocimene and myrcene (Yang et al., 2022). We isolated terpenoid biosynthesis-related genes: *AmDXS*, *AmDXR*, *AmOCS*, and *AmMYS*, and exposed snapdragon flowers with monochromatic blue light and polychromatic light without blue light in order to determine their response to blue light. We found that after 12 h exposure with blue light, the relative expression level of *AmOCS* increased by 7.8-fold, but did not change considerably under dark and EB conditions (Figure 1A). Similar to *AmOCS*, the expression of *AmMYS* significantly increased after blue light exposure compared to dark and EB (Figure 1B). The expression of *AmDXS* and *AmDXR* also increased after blue light exposure (Figures 1C,D). The enhancement of *AmMYS*, *AmDXS*, and *AmDXR* expression was lower than that of *AmOCS*. These results confirmed the effect of blue light to enhance expression of terpenoid biosynthesis-related genes.

### Expression of *AmMYB24* and *AmMYB63* in Response to Blue Light

**FIGURE 2:**
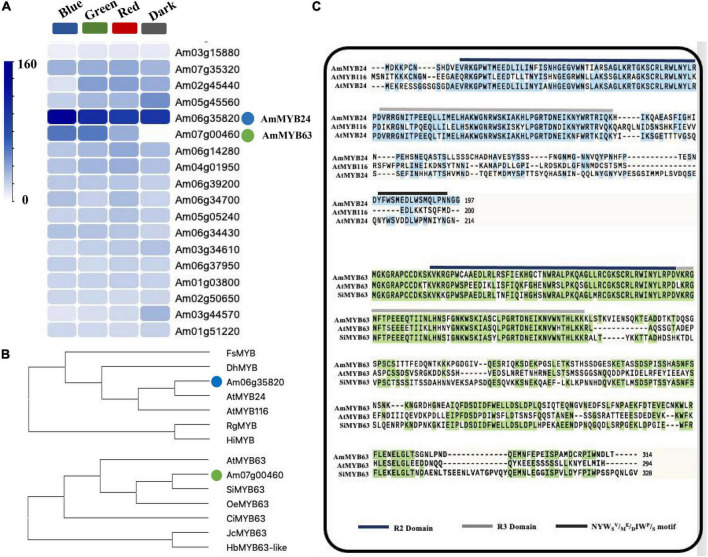
Heatmaps of the expression levels of candidate genes determined by RNA-sequencing and multiple sequence alignment and phylogenetic analysis of *AmMYB24* and *AmMYB63*. **(A)** The FPKM values analysis the candidate TFs from transcriptomic database and illustrated by heatmap. The “blue” circle indicated *AmMYB24*, and the “green” circle indicated *AmMYB63*. **(B)** Phylogenetic tree generated using sequences of *AmMYB63* and *AmMYB24* homologous MYB transcription factors. **(C)** Phylogenetic analysis of *AmMYB63* and *AmMYB24*, R2, and R3 repeats were highlighted in blue and gray colors, respectively. Transactivation domain was highlighted in black.

**FIGURE 3:**
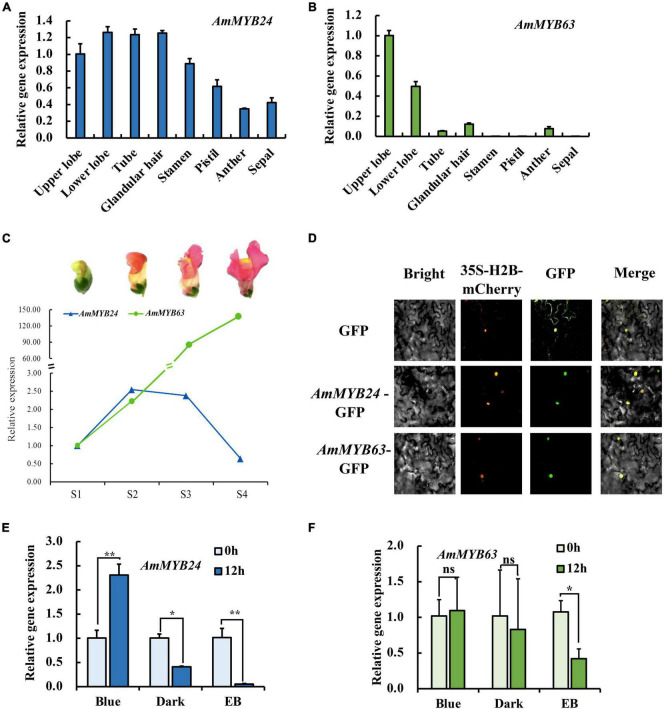
The spatiotemporal expression of *AmMYB24* and *AmMYB63*. **(A)** Expression levels of *AmMYB24* in the different tissues of snapdragon flowers. **(B)** Expression levels of *AmMYB63* in the different tissues of snapdragon flowers. **(C)** Expression profiles of *AmMYB24* and *AmMYB63* in flowers at different developmental stages by qRT-PCR. S1–S4 indicate the flowers at different developmental stages, each stage is separated by 4 days. S1 include flower buds, S2 from 2 to 4 days before flower opening, S3 consisted of flowers from 1 day before anthesis, and S4 consisted of 6 to 8 days old flowers. **(D)** The subcellular localization of *AmMYB24* and *AmMYB63*. Tobacco leaves were transformed with vectors expressing different fusion constructs in combination. Bright, bright field image; 35S-H2B-mCherry, RFP fluorescence nucleus maker detected in the red channel; Green, GFP fluorescence detected in the green channel; Merged, merged GFP and RFP channel images. **(E,F)** Expression profiles of *AmMYB24* and *AmMYB63* under monochromatic blue light (470 nm), polychromatic light without blue light (EB), and dark condition for 0 and 12 h. each experiment was conducted with three biological replicates. The *p*-values were evaluated using the Tukey’s multiple range test. Stars indicate the level of significance, *0.01 < *p* < 0.05, and **p < 0.01.

To investigate the regulatory basis for blue light induction of the expression of terpenoid biosynthesis related genes, we used the results of transcriptome sequencing (Yang et al., 2022) to screen TFs against the reference genome (see text footnote 1) (Li et al., 2019). Two MYB TFs were found from 18 TF genes related to MYB from the RNA-seq data for different light conditions (Figure 2A). These two TF protein sequences include typical R2R3 MYB domains and exhibit high homology to *AtMYB24* and *SiMYB63* (Figures 2B,C), so were named *AmMYB24* and *AmMYB63*, respectively. The expression patterns of these two TFs in snapdragon flowers were detected. Expression of *AmMYB24* was detected in all different parts of snapdragon flowers, with relatively high expression levels in the lower lobe, tube, glandular hair, and upper lobe (Figure 3A). Different from *AmMYB24*, relatively high expression of *AmMYB63* was only found in the upper lobe and lower lobe, and the level in the upper lobe almost twofold higher than that in lower lobe (Figure 3B). Next, the expression levels of these two genes were detected at floral developmental stages from S1 to S4. The transcript expression of *AmMYB24* first increased and then decreased with floral development, with highest levels during S2 and S3 stages. *AmMYB63* showed a gradually increasing pattern in transcript expression from S1 to S4, with the highest level occurred at S4 (Figure 3C). In addition, to determine the site of action of *AmMYB24* and *AmMYB63* in cells, these two TFs were fused with GFP and introduced into *N. benthamiana* leaves. We observed significantly visible fluorescence in the nucleus (Figure 3D), suggesting the specific localization of *AmMYB24-GFP* and *AmMYB63-GFP* fusion proteins in the nucleus, consistent with these two TFs both acting as nuclear regulators. Additionally, both *AmMYB24* and *AmMYB63* showed high expression levels under blue light. Further, after exposure with blue light for 12 h, there was significantly increased expression of *AmMYB24*, with a more than 2.3-fold increase compared with control (Figure 3E). EB condition decreased the expression of *AmMYB24* significantly, to a level far below the expression level under blue light. After 12 h exposure with blue light, there was no significant increase in the relative expression level of *AmMYB63* compared with dark condition (Figure 3F). However, like *AmMYB24*, the expression level of *AmMYB63* was repressed significantly in response to EB (Figure 3F).

### The Regulatory Effects of *AmMYB24* and *AmMYB63* on Terpenoid Biosynthesis-Related Genes

**FIGURE 4:**
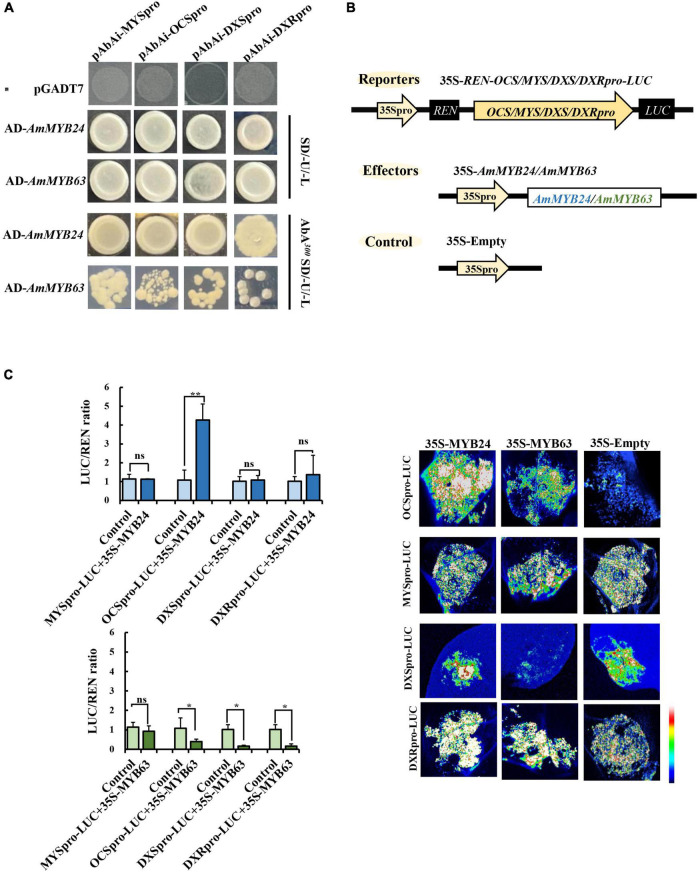
Regulatory roles of AmMYB24 and AmMYB63 on terpenoid biosynthesis-related genes. **(A)** Direct binding of AmMYB24 and AmMYB63 on the promoter and activation of *AmDXS AmDXR*, *AmMYS*, *AmOCS* checked by Y1H. **(B)** Schematic representation of the reporter and effector. Fragments (2,000 bp) of the *AmDXS*, *AmDXR*, *AmMYS*, and *AmOCS* promoters were fused with the LUC gene, the effector plasmid expressed full-length AmMYB24 and AmMYB63 under control of the CaMV 35S promoter. **(C)** The activation effect of AmMYB24 and AmMYB63 on the promoter of *AmMYS*, *AmOCS*, *AmDXS*, and *AmDXR*. The effector, reporter, and control constructs were co-transfected into *Nicotiana benthamiana* leaves, Luciferase (LUC) and REN transcripts were measured and LUC/REN ratio was calculated as described by Liu and Axtell (2015). The results are shown as LUC/REN ratio. LUC imaging showing the activation effect of *AmMYB24* and *AmMYB63* on the promoter of *AmMYS*, *AmOCS*, *AmDXS*, and *AmDXR* in *Nicotiana benthamiana* leaves. each experiment was conducted with three biological replicates. The *p*-values were evaluated using the Tukey’s multiple range test. Stars indicate the level of significance, *0.01 < *p* < 0.05, and **p < 0.01.

**FIGURE 5:**
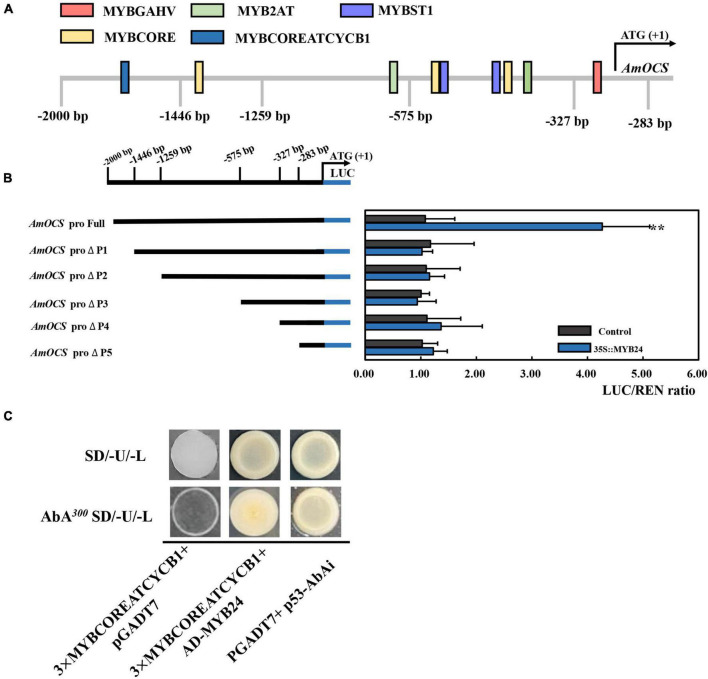
Interaction between *AmMYB24* and the promoter of *AmOCS*. **(A)** The potential MYB binding sites in the promoter of *AmOCS*. The predicted sites are shown with boxes. **(B)** Activation effects of *AmMYB24* on differently truncated *AmOCS* promoters detected by Dul-LUC assays. Each experiment was conducted with three biological replicates. The *p*-values were evaluated using the Tukey’s multiple range test. Stars indicate the level of significance, *0.01 < *p* < 0.05, and **p < 0.01. **(C)** The direct binding of *AmMYB24* to MYBCOREATCYCB1 checked by Y1H. The results are shown as LUC/REN ratio.

To demonstrate the regulatory effects of *AmMYB24* and *AmMYB63*, and determine if these two TFs directly target the terpenoid biosynthesis-related genes, the promoters (2,000 bp sequences) of *AmDXS*, *AmDXR*, *AmOCS*, and *AmMYS* were amplified and cloned to give four reporter vectors, *pAbAiAmDXS*, *pAbAi-AmDXR*, *pAbAi-AmOCS*, and *pAbAi-AmMYS*, respectively. As shown in Figure 4A, the Y1H assay results indicated that both *AmMYB24* and *AmMYB63* could bind to the promoters of *AmOCS*, *AmMYS*, *AmDXS*, and *AmDXR*, suggesting that these two putative TFs interaction with the promoters of these four genes. Based on yeast growth, *AmMYB24* showed better binding affinity for these promoters than that of *AmMYB63*. In addition, the abilities of *AmMYB24* and *AmMYB63* to activate the promoters of *AmOCS*, *AmMYS*, *AmDXS*, and *AmDXR* were also performed by using a dual-LUC assay in *N. benthamiana* leaves. The reporter vectors were generated, and *AmMYB24* and *AmMYB63* were cloned into pNC-MCS35S vectors (Figure 4B) and co-transfected into *N. benthamiana* leaves (Figure 4C). The LUC/REN ratios revealed that *AmMYB24* only activated *AmOCS* promoter *in planta* (Figure 4C). Differently, *AmMYB63* represses the activities of the four promoters. Therefore, *AmOCS* was selected for the further validation of the regulatory effect. In the *AmOCS* promoter sequence, nine MYB binding sites (MBS) were identified using SOGO online (see text footnote 2), including two MYB2AT motifs, three MYBCORE motifs, two MYBST1 motifs, one MYBCOREATCYCB1 motif, one MYBGAHV motif (Figure 5A). To determine the binding site of *AmMYB24* in the *AmOCS* promoter, five reporter vectors containing the LUC reporter gene driven by differently truncated versions of the *AmOCS* promoter were constructed (Figure 5B). Through dual-LUC assays, we found that there was no significant difference in the LUC/REN ratio when the promoter was truncated to *P1*, *P2*, *P3*, *P4*, or *P5*, but significantly higher LUC activity was detected when the promoter was full-length upstream of the ATG start codon, a level that was about four times higher LUC activity than control (Figure 5B). This was tested further by Y1H assay, which indicated that the sole MYBCOREATCYCB1 motif (TAACAAA) between positions −1,446 and −2,000 bp upstream of the ATG is most likely the target of binding of *AmMYB24* to the *AmOCS* promoter (Figure 5C). These results revealed that *AmMYB24* acts as a regulator of *AmOCS* by directly binding to the promoters of terpenoid biosynthetic genes, particularly *AmOCS*.

### Effect of Overexpressing and Silencing *AmMYB24* on Ocimene Biosynthesis

**FIGURE 6:**
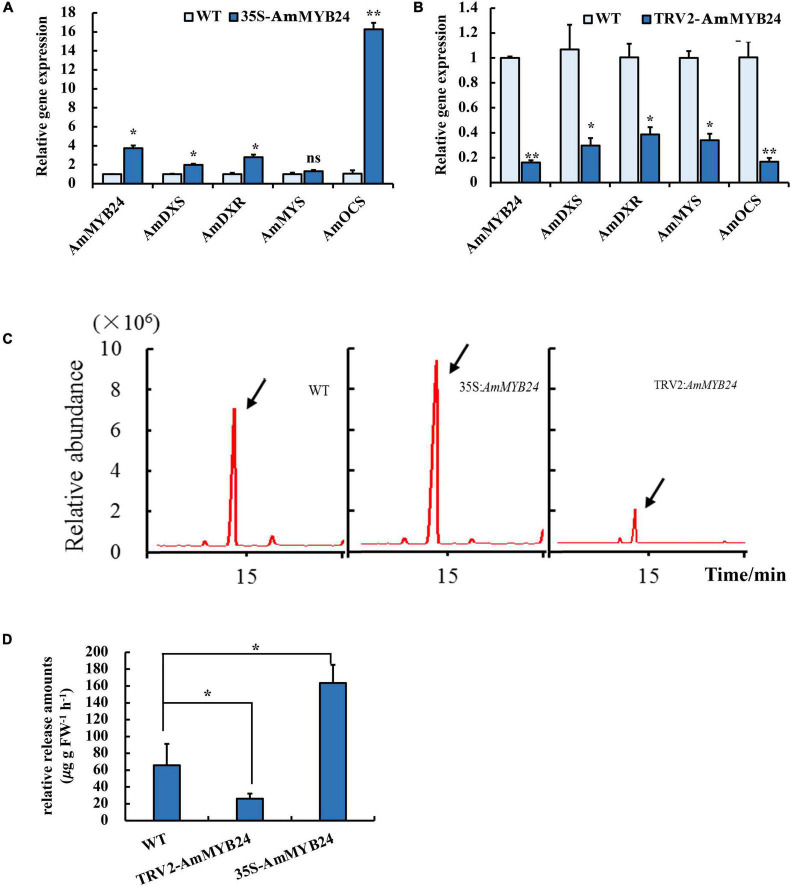
Effect of overexpression and silencing *AmMYB24* on terpenoid biosynthesis. **(A)** Relative expression of *AmMYB24*, *AmDXS*, *AmDXR*, and *AmOCS* after transiently overexpressing *AmMYB24*. **(B)** Expression levels of *AmDXS AmDXR*, *AmMYS*, *AmOCS*, and *AmMYB24* after transiently silencing *AmMYB24*. **(C)** The total ion chromatogram (TIC) of ocimene after overexpressing and silencing *AmMYB24*, peaks marked with arrow represent the ocimene produced by *AmOCS*. **(D)** The release amounts of ocimene after overexpressing and silencing *AmMYB24*. each experiment was conducted with three biological replicates. The *p*-values were evaluated using the Tukey’s multiple range test. Stars indicate the level of significance, *0.01 < *p* < 0.05, and **p < 0.01.

To validate the function of *AmMYB24* in ocimene biosynthesis, the expression levels of *AmMYS*, *AmDXS*, *AmDXR*, and *AmOCS* and the emission of ocimene were investigated after transiently overexpressing and silencing *AmMYB24* in snapdragon flowers. In snapdragon flowers overexpressing *AmMYB24*, the expression of *AmMYB24* increased to about fourfold the expression level of WT, and *AmOCS* expression was remarkably up-regulated by 15-fold compared with the WT expression level (Figure 6A). The expression levels of both *AmDXS* and *AmDXR* were also enhanced by overexpressing *AmMYB24* (Figure 6A). As expected, silencing *AmMYB24* resulted in a significant decrease in the expression level of *AmOCS*, to a level about 1/5 that of WT (Figure 6B). As per Figure 6B, reduction in the expression levels of *AmOSC*, *AmDXS*, *AmDXR*, and also *AmMYS* in *AmMYB24* silenced flowers. An obvious increase and decrease in the emission of ocimene was observed by TIC in flowers with *AmMYB24* overexpression and silencing, respectively (Figure 6C). *AmMYB24* overexpression resulted in a twofold increase in ocimene release compared with WT, and a 70% decrease in ocimene release was found in *AmMYB24* silenced flowers (Figure 6D).

### Interaction Between AmMYB24 and AmCRY1

**FIGURE 7:**
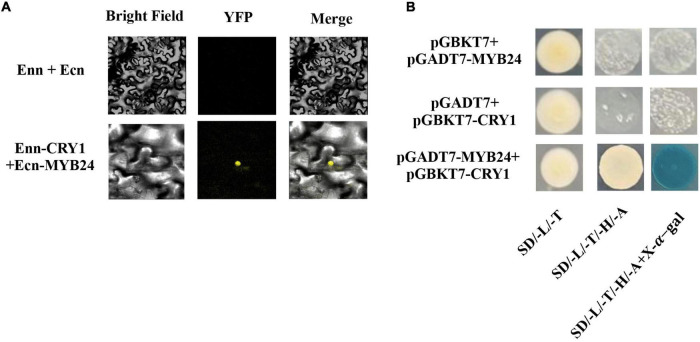
Interaction between AmMYB24 and AmCRY1. **(A)** Y2H assay to test the interactions of AmCRY1 with AmMYB24. AmCRY1 was fused in pGBKT7, and *AmMYB24* was fused with the activation domain (AD) in pGADT7. Interactions (represented by blue color) were assessed on SD/-Ura/-His/-Trp/-Leu/X-α-Gal medium. **(B)** BiFC assay to detect the interactions of AmMYB24 with AmCRY1. *AmMYB24* was fused with the pNC-BiFC-Ecn, and *AmCRY1* was fused with pNC-BiFC-Enn. YFP fluorescence was detected 48 h after co-expression of the indicated construct pairs in leaves of *Nicotiana benthamiana*.

**FIGURE 8:**
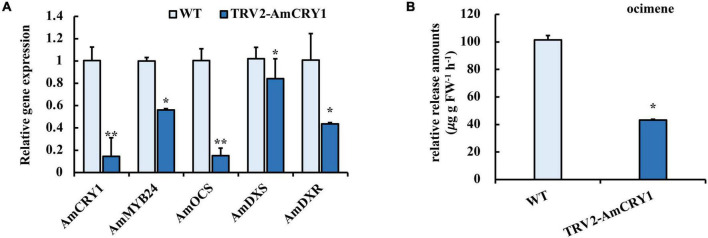
Effect of silencing *AmCRY1* on ocimene biosynthesis. **(A)** Expression levels of *AmCRY1*, *AmMYB24*, *AmOCS*, *AmDXS*, and *AmDXR* in *AmCRY1* silencing flowers. **(B)** Release amount of ocimene in *AmCRY1* silencing flowers. each experiment was conducted with three biological replicates. The *p*-values were evaluated using the Tukey’s multiple range test. Stars indicate the level of significance, *0.01 < *p* < 0.05, and **p < 0.01.

To identify upstream signaling events, we next investigated the potential role of CRY1 in the blue light induction of ocimene. An interaction between AmMYB24 and AmCRY1 was detected using Y2H assays (Figure 7B). Additionally, BiFC assay was used to assay the interaction between these two proteins (Figure 7A). The yellow fluorescence signals indicated interaction between AmMYB24 and AmCRY1 in *N. benthamiana* leaves. To validate the function in ocimene biosynthesis, after silencing *AmCRY1* by VIGS, effects on gene expression and ocimene emission were detected. We found that the expression levels of *AmMYB24*, *AmOCS*, and *AmDXR* were reduced significantly by 60–90% in *AmCRY1*-silenced flowers. (Figure 8A). The emission of ocimene was also inhibited in *AmCRY1* silenced flowers, with a 60% decrease in ocimene production (Figure 8B). These findings indicated that *AmCRY1* can mediate ocimene biosynthesis by interacting with *AmMYB24* in snapdragon flowers.

## Discussion

Floral volatiles are important secondary metabolites for flowering plants to attract pollinators and predators, and enhance ornamental and economic value (Vranova et al., 2013). The emissions of floral volatiles are impacted by light qualities. In our previous study, blue light promoted floral terpenoid emissions from snapdragon (Yang et al., 2020), but the regulatory mechanism was not disclosed. So, we investigated how blue light regulated terpenoid biosynthesis.

Compared with red and green light, blue light enhanced the emission of ocimene and myrcene by activating the expression of *AmOCS*, *AmMYS*, *AmDXS*, and *AmDXR*. By comparison of expression levels, we confirmed induction of *AmOCS*, *AmMYS*, *AmDXS*, and *AmDXR* by blue light, with a more significant effect on *AmOCS* than on *AmMYS*, *AmDXS*, and *AmDXR*. The results suggested that blue light might not enhance the expression levels of all the genes in MEP pathway, but some important node genes (Yang et al., 2020). In postharvest *C. sinensis* leaves, blue light significantly increased most endogenous volatiles including volatile terpenes by up-regulating the expression levels of terpene synthases (Fu et al., 2015). In *Arabidopsis*, light regulates the expression of *AtTPS03* for terpenoid biosynthesis (Michael et al., 2020). DXS and DXR were identified as key committed enzymes for floral terpenoid volatiles (Zhang et al., 2018), and light can induce expression of *Arabidopsis DXS* and *DXR* genes (Carretero-Paulet et al., 2002; Hsieh and Goodman, 2005). Red light exposure increased expression of two *Arabidopsis TPS* genes compared to dark condition (Hong et al., 2012). Thus, the effects of light qualities on the terpenoid pathway may vary for diverse plants, and the differences may be related to upstream regulation of terpenoid biosynthesis. Moreover, by comparing the expression in response to blue light and full wavelength light without blue light, it was found that the expression of *AmOCS* gene was most affected by blue light (Figure 1A), indicating that *AmOCS* is the terpenoid biosynthesis gene regulated by blue light in snapdragon flowers.

Transcription regulation plays an important role in terpenoid biosynthesis (Hong, 2016). We analyzed transcriptome data and identified two R2R3 MYB TFs, *AmMYB24*, and *AmMYB63*, which exhibited high expression levels under blue light (Figure 2A), similar to the changes observed in terpenoid emission and biosynthesis gene expression. This suggested a potential relationship between these two TFs and terpenoid biosynthesis. Cloning and expression analysis revealed that compared to *AmMYB63*, *AmMYB24* exhibited a pattern that was more similar to those reported for ocimene and myrcene emission in snapdragon (Figures 3A,B; Dudareva et al., 2003). Transcriptional regulation of MYB on volatile biosynthesis must be achieved by binding the promoters of key structural genes. We used yeast-one hybrid and dual-LUC assays and found that AmMYB24 can directly bind to the promoters of *AmDXS*, *AmDXR*, *AmMYS*, and *AmOCS*, with the strongest activation seen with *AmOCS* (Figure 4C). MYBs regulate target gene expression by recognizing and binding specific promoter motifs. The MYBCOREATCYCB1 motif (TAACAAA) was identified in the promoter of *AmOCS* as the binding site of AmMYB24, playing a key role in *AmMYB24*-mediated transcriptional regulation of *AmOCS* (Figure 5). There are differences and considerable similarities in sequence binding specificity for MYBs, especially for members of a single phylogenetic branch (Romero et al., 1998). Different from our results, in *F. hybrid*, FhMYB21L2 directly binds the MYBCORE motif of *FhTPS1*, and AtMYB21 specifically binds the MYBCORE motif in both *AtTPS14* and *AtTPS21* promoters in *Arabidopsis* (Yang et al., 2020). Furthermore, AmMYB24 function was confirmed to play a pivotal role in ocimene biosynthesis by *in vivo* transient overexpression and silencing of the *AmMYB24* gene in snapdragon flowers (Figure 6). Overall, the results confirmed that AmMYB24 can activate *AmOCS via* binding to the MYBCOREATCYCB1 motif in the promoter to regulate the biosynthesis of floral terpenoid volatiles.

A blue light receptor, CRY1, has been reported to mediate photoresponses in plants, playing a pivotal role in plant growth and development (Yu et al., 2010). To explore the upstream signaling pathway of blue light induction of the biosynthesis of floral terpenoid volatiles, the role of CRY1 was investigated. An interaction between AmCRY1 and AmMYB24 was demonstrated by Y2H and BiFC assays (Figure 7). After silencing *AmCRY1*, the expression of *AmMYB24* and terpenoid biosynthesis genes were repressed, and the terpenoid emission was also decreased (Figure 8). This confirmed the importance of AmCRY1 in the induction of terpenoid biosynthesis by blue light. In *Artemisia annua*, overexpression of *CRY1* elevated transcript levels of genes encoding enzymes involved in sesquiterpene, resulting in increased accumulation of sesquiterpene (Hao et al., 2019; Shen Q. et al., 2016). For blue light, two classical pathways of CRY signal transduction have been discovered: SUPPRESSOR OF PHYA1/CONSTITUTIVELY PHOTOMORPHOGENIC1 (SPA1/COP1)-dependent CRY regulation of proteolysis, and CRY interacting bHLH1 (CIB1)-dependent CRY2 regulation of transcription (Wang et al., 2018). In the second pathway, ELONGATED HYPOCOTYL 5 (HY5) usually acts as the downstream TF of COP1 to regulate the light-dependent response (Ang et al., 1998). In this study, we found that AmCRY1 can interact directly with MYB TF, AmMYB24, to regulate its transcriptional activity on terpenoid biosynthesis. A direct interaction of CRY1 and a TF, HBI1 (HOMOLOG OF BEE2 INTERACTING WITH IBH 1) was previously shown to lead to inhibition of HBI1’s DNA binding activity and target gene expression (Wang et al., 2018). It is likely that additional TFs that interact directly with CRY will be discovered. TFs can also be regulated by HY5. In light-regulated flavonoid biosynthesis, MYB12 is a HY5-dependent light-inducible gene that plays a key role in the activation of the flavonol biosynthesis in response to light (Bhatia et al., 2021). MdWRKY41 is a downstream target of MdHY5, and regulates anthocyanin and proanthocyanin accumulation (Mao et al., 2021). Additionally, MYB can act with COP1 to manage anthocyanin biosynthesis in response to light (Maier et al., 2013). Whether AmMYB24 can directly interact with HY5 or COP1 in floral terpenoid biosynthesis was not tested here, and should be elucidated in future study.

**FIGURE 9:**
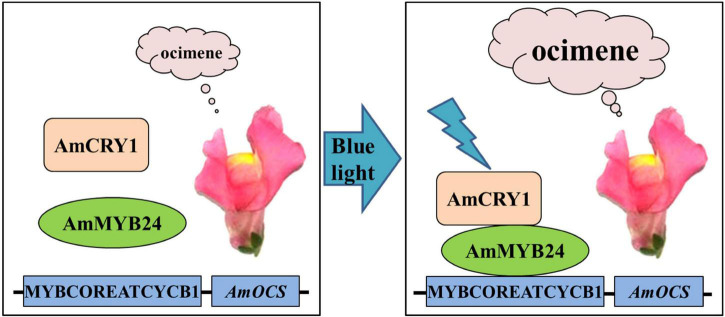
Proposed regulatory model mediated by AmCRY1 and AmMYB24 in the ocimene biosynthesis induced by blue light. When snapdragon flowers are exposed to blue light, AmCRY1 is first activated, and the light signal is then transduced to AmMYB24 through interaction with AmCRY1. AmMYB24 would subsequently bind to the MYBCOREATCYCB1 motif and induce *AmOCS* expression, which finally results in the massive ocimene emission of blooming flowers.

In conclusion, we demonstrated that AmMYB24 and AmCRY1 contribute to the induction of terpenoid biosynthesis by blue light, and proposed a potential regulatory pathway of ocimene biosynthesis in Snapdragon (Figure 9). When snapdragon flowers are exposed to blue light, AmCRY1 is first activated, and then the light signal can be transduced to AmMYB24 through interaction with AmCRY1. AmMYB24 then activates *AmOCS* by binding to its MYBCOREATCYCB1 motif, which triggers abundant ocimene emission. Given the diversity and complexity of the light transduction pathway, the details of the mechanism by which light quality can regulate terpenoid biosynthesis require further investigation.

## Data Availability Statement

The datasets presented in this study can be found in online repositories. The names of the repository/repositories and accession number(s) can be found below: National Center for Biotechnology Information (NCBI) BioProject database under accession number: PRJNA811724.

## Author Contributions

JH performed the experiments and wrote the manuscript. TL, XW, XZ, XB, HS, and SW helped with data analysis. ZH analyzed the results. JW and PL designed the experiments and supervised the research work. All authors contributed to the article and approved the submitted version.

## Conflict of Interest

The authors declare that the research was conducted in the absence of any commercial or financial relationships that could be construed as a potential conflict of interest.

## Publisher’s Note

All claims expressed in this article are solely those of the authors and do not necessarily represent those of their affiliated organizations, or those of the publisher, the editors and the reviewers. Any product that may be evaluated in this article, or claim that may be made by its manufacturer, is not guaranteed or endorsed by the publisher.

## Footnotes

1
http://bioinfo.sibs.ac.cn/Am/index.php

2
http://sogo.dna.affrc.go.jp/
