# Supplementary material for: AmMYB24 Regulates Floral Terpenoid Biosynthesis Induced by Blue Light in Snapdragon Flowers
Source: Front Plant Sci. 2022 Jul 1;13:885168. doi: 10.3389/fpls.2022.885168 (PMC9284265; doi:10.3389/fpls.2022.885168)
Supplement: Supplementary file 1 [file Table_1.DOCX]

Supplementary Material

**Table1. primers sequences used in this study**

|  | Primer name | Primer sequence5’-3’ | Primer sequence5’-3’ |
| --- | --- | --- | --- |
| For Dual-LUC essay | *FLUC-F*  *RLUC-F* | GTTTTGGAGCACGGAAAGAC  TGTTGGACGACGACGAACTTCAC | CAAGAGTAAAAGATAGTAAAACCGG  CATTTTTGTCGGCCATGATT |
| For TFs cloning | *MYB24*  *MYB63* | AATTTCTAACTTTTTGGAAACACGT  ATGGGTAAAGGCCGTGCA | CCATCTTAATTGGTAGAGAGAGAAAG  CTAAGTTAAGTCATTCCATATTGGGC |
| qRT-PCR | *MYB24*  *MYB63*  *MYS*  *OCS*  *DXS*  *DXR*  *UBI*  *CRY1* | CCAGATGTGAGAAGGGGAAA  GTGTGCTGCTGAGGATTTGA  GTCATAACACGAGTAAACTACATC  TGCCCGACAACATGAAGA  ACGTTTCCCATTTCTTGTGC  CAATGTTTGGCACAGACACC  AGCCGATGGAAGTATATGTTTGGACATC  CACTGGTTCGCGATCACAAA | TGGCTTCAGCTTGTTTGATG  TCTTCCTCAGGCGTGAAGTT  AGTGCCTGATGAAGTTTGGA  ATACTCGTTGGCGCTTGG  CTGTCCTTGCCTTCACCAAT  GCAAGTGCCACAACTCTGAA  CTAACTTTGCGGTTATAATCTCGTTTA  CTGTCCCAAAATCCGGCAAA |
| For promoter cloning | *OCS*  *MYS*  *DXS*  *DXR*  *MYBCOREATCYCB1* | CCATACATTTTAACTTGCCGTCTAA  AGCGAGGAAGCGGAG  CCAATACTCGTGGTATATTCATGA  CAATACTCCCACGCAGCCCACGCG  TGGCCGTTCAGTGGCCGTTCAGTGGCCGTTCAG | TTCTAGTGTGCATTTTAAACGTTTT  GACGAATGATATTTGTGTATGATACG  AGAAGCCATTTCTCTTTCTTATTTT  CGGGGAGAGCATATTCAACGCCAT  CTGAACGGCCACTGAACGGCCACTGAACGGCCA |
| For promoter truncation | *OCS(-307)*  *OCS(-346)*  *OCS(-497)*  *OCS(-597)*  *OCS(-908)*  *OCS(-1281)*  *OCS(-1468)* | ggtctcagcagaccacaagtTTCTAGTGTGCATTTTAAACGTTTT  ggtctcagcagaccacaagtTTCTAGTGTGCATTTTAAACGTTTT  ggtctcagcagaccacaagtTTCTAGTGTGCATTTTAAACGTTTT  ggtctcagcagaccacaagtTTCTAGTGTGCATTTTAAACGTTTT  ggtctcagcagaccacaagtTTCTAGTGTGCATTTTAAACGTTTT  ggtctcagcagaccacaagtTTCTAGTGTGCATTTTAAACGTTTT  ggtctcagcagaccacaagtTTCTAGTGTGCATTTTAAACGTTTT | agtggtctctgtccagtcctAATAGCTAGCCAAGATTTTTTTTTG  agtggtctctgtccagtcctGTTGTAGCAAAACACGTGAA  agtggtctctgtccagtcctCGTGCTTGATTGAATTAGGG  agtggtctctgtccagtcctAAAGAAATTAACTTACGCTGCAT  agtggtctctgtccagtcctGTAAAAAGTAGCAGCTTTCACT  agtggtctctgtccagtcctGCACTTTCTTGAGTTCAATTACT  agtggtctctgtccagtcctACGCTTATTAAACTGTCAATTGA |
